# A Burgeoning Crisis? A Nationwide Assessment of the Geography of Water Affordability in the United States

**DOI:** 10.1371/journal.pone.0169488

**Published:** 2017-01-11

**Authors:** Elizabeth A. Mack, Sarah Wrase

**Affiliations:** 1 Department of Geography, Environment & Spatial Sciences, Michigan State University, East Lansing, Michigan, United States of America; 2 Department of Accounting & Information Systems, Eli Broad College of Business, Michigan State University, East Lansing, Michigan, United States of America; Stony Brook University, Graduate Program in Public Health, UNITED STATES

## Abstract

While basic access to clean water is critical, another important issue is the affordability of water access for people around the globe. Prior international work has highlighted that a large proportion of consumers could not afford water if priced at full cost recovery levels. Given growing concern about affordability issues due to rising water rates, and a comparative lack of work on affordability in the developed world, as compared to the developing world, more work is needed in developed countries to understand the extent of this issue in terms of the number of households and persons impacted. To address this need, this paper assesses potential affordability issues for households in the United States using the U.S. EPA’s 4.5% affordability criteria for combined water and wastewater services. Analytical results from this paper highlight high-risk and at-risk households for water poverty or unaffordable water services. Many of these households are clustered in pockets of water poverty within counties, which is a concern for individual utility providers servicing a large proportion of customers with a financial inability to pay for water services. Results also highlight that while water rates remain comparatively affordable for many U.S. households, this trend will not continue in the future. If water rates rise at projected amounts over the next five years, conservative projections estimate that the percentage of U.S. households who will find water bills unaffordable could triple from 11.9% to 35.6%. This is a concern due to the cascading economic impacts associated with widespread affordability issues; these issues mean that utility providers could have fewer customers over which to spread the large fixed costs of water service. Unaffordable water bills also impact customers for whom water services are affordable via higher water rates to recover the costs of services that go unpaid by lower income households.

## Introduction

While basic access to clean water is critical, another important issue is the affordability of water access for people around the globe. In terms of water affordability, studies have estimated that approximately 60% of the population in low-income countries could not afford water priced at full cost recovery rates[1]. This is problematic given that consumers do not pay the full cost of water because water is subsidized by the government in many countries or priced at below cost-recovery levels. While research has highlighted issues with access to affordable water in developing countries and some developed countries 2–4], more work is needed in developed countries to better understand the extent of this issue in terms of the number of households and persons impacted. In cities across the United States, water affordability is becoming an increasingly critical issue. Mass shutoffs in Detroit, Michigan have resulted in the termination of service for 50,000 households since the start of a campaign in 2014 to shut off water for delinquent residents[4]. In Philadelphia, Pennsylvania an estimated 227,000 customers, or 4 out of 10 water accounts, are past due [5]. Atlanta, Georgia and Seattle, Washington have some of the highest water rates in the country at $325.52 and $309.72 per month for a family of four, respectively [6]. These rates are based on 100 gallons (378.54 liters) of water per person per day including, water, sewer, and storm water for 5/8 inch (15.875 mm) meters [7]. It is likely these rates will rise as the cost of providing water increases.

A variety of pressures ranging from climate change, to sanitation and water quality, to infrastructure upgrades, are placing increasing strain on water prices[8]. Estimates of the cost to replace aging infrastructure in the United States alone project over $1 trillion dollars are needed in the next 25 years to replace systems built circa World War II, which could triple the cost of household water bills [9]. Other studies estimate that adaptations to water systems to deal with climate change will cost the United States more than $36 billion by 2050 [10]. For example, an increasing number and intensity of weather events call for improvements to waste-water facilities to manage storm water [10]. A hidden pressure on urban water systems is slow or even declining population growth, which reduces the customer base over which the high fixed costs of water services are distributed, and places increasing pressure on household water bills. This is the case in Detroit where a declining population has left fewer customers to pay for water. Another critical issue is the suburbanization of people, which also leaves providers in core central city areas with fewer customers to pay for water services. This means that for cities across the United States, shrinking populations in particular metropolitan areas and in downtown areas, combined with other pressures on urban water systems, present a perilous future for water utilities and their customers [11].

Over the next few decades, water prices are anticipated to increase to four times current levels [8]. Prices could go higher if cities look to private providers for water services, who have a tendency to charge higher rates than public providers [12–15]. These pressures on water systems, combined with the fact that water is a vital necessity to sustain life, place this issue at the forefront of 21^st^ century infrastructure challenges. While studies have found that Americans are willing to pay more to maintain and ensure access to water resources [8], this willingness to pay may conflict with their fundamental ability to pay for water. To date however, work on water affordability for low income households in developed countries has received somewhat less attention than work on water in the developing world [3]. International work on affordability [3,2,16,17] and case studies in the United States have highlighted specific communities where affordability is an issue [10,18]. While valuable, the extent that water affordability is a widespread issue for U.S. households, and where these households are located, remains unclear. This is vital to unravel since there is currently no federal statute or policy that ensures water access for poor residents [19].

Given this gap in work on water issues, the goal of this paper is to assess the extent that the ability to pay for water services is a potential issue for U.S. households. This assessment will create a benchmarking tool, based on EPA affordability standards, to provide a means of assessing the potential financial capacity of households to pay for water service. It will also assess the characteristics and geographic locations where residents are potentially susceptible to rising water rates. Results of this assessment highlight a possible affordability crisis in which an estimated 11.9% of households could find current water prices unaffordable; this percentage could triple to 35.6% if rates increase according to recent projections.

## Assessing and Measuring Water Affordability

Affordability is not only an issue for customers in the United States but for utility customers around the globe. This is the case for low-income households who must use a disproportionate share of income to pay for water services [3,2]. An affordability study in France highlights that single parent families, particularly female-headed households, and large households receiving social assistance are most at risk for affordability issues [20]. In many countries, water is also not priced at full cost recovery levels, which means that this affordability challenge is likely to worsen in the near future. For example, in several countries in Africa where the cost of infrastructure is subsidized by the state or donors, customers do not pay the full cost of water [1]. However, it is important to note that affordability issues are highly contextual and depend on the country analyzed, as well as the spatial scale of the analysis. A recent study of Tunisia found for example, that water rates were affordable and could sustain some form of price increase [21]. Water rates are also highly variable within countries and major metropolitan areas [22]. This means that affordability studies must be interpreted with some caution since results from one study area are likely not applicable to a range of consumers in other geographic locations and water provision contexts.

### Measuring Water Consumption

Aside from the variability of water rates at a variety of spatial scales, the extent that water rates are deemed affordable depends on the water consumption level analyzed and the affordability benchmark used. In this regard, there is a wide range of consumption levels and benchmarks to consider. A variety of studies have highlighted that water consumption varies within and between countries [23,24], and that this variation is related to several characteristics of people and places ranging from climate lifestyle, diet, and income [25]. For example, Gleick [23] highlights that water use within the state of California alone varies between 42.17 gallons (171 liters) and 140.28 gallons (531 liters) per person per day. A more recent study highlights tremendous variation in water use within developed countries. Uruguay, for example, uses 16.11 gallons per person per day (61 liters per person per day (1/c/d)) while Canada uses 203.15 gallons per person per day (769 1/c/d) [24].

A good portion of these studies have attempted to unravel the minimum level of water consumption to meet human needs, with varied results. Gleick [23] suggests that the minimal level of water consumption is about 13.21 gallons (50 liters) per person per day. This is the equivalent of 396 gallons (1499.02 liters) per person per month. A more recent study estimates however that people need on average 35.66 gallons per person per day (135 1/c/d), which is over twice the recommended amount [23]. Determining basic water needs is challenging not only because of the unique climatic and cultural characteristics of place [25], but also because it is difficult to disentangle essential uses of water from non-essential uses [16]. In fact, there are several non-essential uses of water such as swimming pools, extra showers, and outdoor watering that raise average consumption levels across countries [16]. Perceptions of “essential” uses are likely to vary dramatically based on the income, culture, religion, and diet of people. A classic example of this is water use for personal hygiene. In several countries around the world, flushing toilets are a rarity while in other countries they are quite common, if not ubiquitous. Thus, the extent that flushing toilets are considered a hygienic necessity will influence the perception of baseline or essential uses of water.

### Measuring and Benchmarking Affordability

Aside from variations in what is considered “essential” water consumption, affordability analyses are complicated by the variety of methods for quantifying affordability, and the variety of benchmarks used to assess affordability. That said, there are two basic approaches to quantifying affordability: expenditure based measures and income based measures [3]. Within these two categories, Hutton [26] highlights four affordability metrics: 1. Expenditures as a share of household income 2. Cost of equipment needed to access water 3. All water related expenditures as a percentage of household income and 4. Full financial and economic costs as a percentage of annual income. Each of these metrics has its advantages and disadvantages that stem from the ease of obtaining the data, to the comprehensiveness of the metrics. For example, expenditure data are generally easier to obtain, but do not necessarily cover the full cost of accessing and using water [26]. A consideration of the full financial and economic costs of water is more comprehensive, but is data intensive and involves the difficulties of measuring “true” economic costs [26].

In terms of expenditure-based compared to income-based measures, some studies suggest that expenditures provide a better sense of affordability because household income data do not include all sources of household revenue, particularly in developing countries with a large informal economy [3]. One of the primary issues with expenditure-based approaches is that households with average or above average incomes, who consume large amounts of water for non-essential purposes, may be deemed water poor [16]. This is quite a distinct situation from low-income households who might be classified as water poor, but whose water consumption is dedicated to essential uses of water only.

Just as there are quite a few ways of measuring water affordability, so too are there a variety of benchmarks used to assess the relative affordability of water. Income-based benchmarks are more common than are expenditure-based benchmarks, which is demonstrated by the number of income-based affordability benchmarks used by several organizations. For example, the affordability standard adopted by the United Nations Development Program and the United Kingdom’s Department of the Environment, Transport, and the Regions (DETR) is 3% of household income. This benchmark is similar to the OECD recommendation that household water bills not consume more than 3–5% of household income [27,28]. The Unitary Universalist Service Committee advocates for 2.5% of monthly household income [10,26,29] which is similar to the U.S. Environmental Protection Agency (EPA) standard that households spend no more than 2% on water and 4.5% of median household income on both water and wastewater services [30]. The U.S. EPA threshold of 4.5% is similar to the World Bank income benchmark of 5% [1]. Although less popular than income-based benchmarks, an example of an expenditure-based affordability benchmark is 5% of household expenditures for water and wastewater services [3]. Given this range of affordability benchmarks, this study adopts the U.S. EPA’s income-based standard for measuring water affordability which states that combined water and wastewater services should comprise no more than 4.5% of median household income. This combined measure is used because water and wastewater services appear together on customer bills and because of the rising costs of wastewater treatment, which comprise a growing portion of water bills [6].

## Pricing Water

Providing water service involves a large amount of funds to deploy and maintain infrastructure. These funds are typically divided into two categories: 1. operations and 2. maintenance and capital improvements [31]. The second of these components covers the costs of new equipment, new facilities, infrastructure maintenance and rehabilitation costs to meet new regulatory requirements, such as those outlined in the Safe Drinking Water Act (SDWA), [31]. The goal in pricing water is to set rates that are low for consumers but also sufficient to recover the large fixed costs of building and maintaining infrastructure. A key facet of pricing water rests on the ability to achieve declining average costs. This means that the cost per consumer falls the greater the number of customers over which fixed costs can be distributed. If either the number of customers falls and/or the amount of fixed costs increases, water rates rise.

Aside from the cost of providing service, another driver of water rates is the characteristics of the utility provider. In this regard, two characteristics, provider size and public or private orientation are important to consider. Large providers serve populations of 100,000 or more while small providers serve populations of 10,000 or fewer [32]. The size of provider is important to consider because recent research suggests that smaller providers face a variety of challenges including diminishing customer bases, fewer financial resources, and a lack of engagement in long-range planning [32]. In addition to provider size, the public or private orientation of providers also factors into water rates. In the United States, private entities can make a profit on water services while public providers are required to price water on a cost-recovery basis [12]. This means government-owned utilities are responsible for setting rates while investor-owned utilities set rates via water rate cases [33]. In both the government and investor-owned utility situations, the public plays a role in setting rates [33]. Private providers, however, set rates differently and are not mandated to restrict rates to cost recovery alone. Thus, several studies find that the rates of private entities exceed those of public providers [12–15]. While the majority of U.S. consumers are currently serviced by public providers [12], a growing proportion of water services in the U.S. may be provided by private providers in the near future, as U.S. cities grapple with a lack of funding for infrastructure. From this perspective, privatization offers the option to earn profits on water services, which may be used to fund other initiatives and balance city budgets [13].

From an affordability perspective, all of these pressures on water resources and infrastructure forebode rising water rates for consumers. However, the implications of these rate increases on the affordability of water services for U.S. households remain unassessed on a national scale. Instead, much of the work on affordability has emphasized the perspective of water providers [8,34–36],. Although this body of work acknowledges affordability issues for low-income households [8,34] more work is needed to assess who is impacted and where impacted households are located.

## Methodology

In the United States, there is no national database about water prices at the community level. This makes national level assessments of affordability challenging. If information about water prices is needed, there are two ways to obtain it. The first way is to scrape price information from provider websites or call providers to get pricing information. A 2010 study of water prices in the Great Lakes region used this approach to collect sample data on water rates [37]. The second approach, which is used frequently in water pricing analyses [38] is to use information from the American Water Works Association (AWWA) biennial survey of water and wastewater rates.

AWWA is the largest non-profit association in America dedicated to research and education about water management and treatment [39]. The data used in the present affordability study are derived from a 2015 survey of 318 AWWA member utilities in the United States, Canada, and Puerto Rico [22]. In this survey, 211 providers (66%) report information about combined water and wastewater costs and 90 providers (28%) report information about combined water, wastewater and storm water costs. Table 1 provides a breakdown of the 296 providers reporting information about their year-round residential rate structure.

**Table 1:** Rate Structure Breakdown of AWWA Reporting Providers.

Rate Structure	Number of Reporting Providers	Percent of Reporting Providers
Increasing block	147	50%
Decreasing block	48	16%
Increasing-decreasing block	11	4%
Uniform volumetric	86	29%
Flat rate	4	1%
Total	296	100%

Source: American Water Works Association (AWWA) 2015

Aside from rate information, the survey collects a wide range of characteristics about retail and wholesale providers including: location and municipalities served, sales volume, source of water, size of the provider and ownership model [22]. Of all the content in this survey, information from the median household affordability section of the summary report was of primary importance. In this section of the report, there is information about median household income for the service area of 187 utility providers, as well as charges as a percentage of median household income (MHI) for various water consumption tiers; these tiers correspond to 3,740 gallons/month (14,157.44 liters) 7,480 gallons/month (28,314.88 liters), 11,220 gallons/month (42,472.32 liters), and 22,400 gallons/month (84,793.22 liters) [22]. From this information, it was possible to compute the annual water bill in 2014 dollars for a residential customer for each consumption tier. The formula for this calculation is as follows:

Annual water bill=% MHI for monthly consumption level × MHI (1)

After obtaining the annual water bill for each consumption tier for each provider, the per gallon cost of water was computed for each consumption level (S1 Table). This was necessary for two reasons, one, to evaluate whether the unit cost of water is associated with the water consumption level. Two, to compute the annual cost of water for water consumption levels outside those provided in the AWWA survey. For example, the EPA considers average consumption to be 12,000 gallons (45,424.94 liters) per month for a household of 4 people [40]. The general formula for this calculation is as follows:

Unit cost=Annual water bill (by consumption level)/(Annual water consumption in gallons) (2)

After computing this per gallon cost for combined water and wastewater service at each consumption level, these unit costs were averaged to obtain a per unit cost for a gallon (liter) of water. The unit cost was $0.01 for one gallon of water ($0.00264 per liter). This number includes service fees for both water and wastewater services and did not vary according to the volume of water consumed. Aside from this flat per unit price across consumption levels, an average cost is also used because conversations with utility managers revealed this is industry practice for comparing the cost of water services across different providers.

Based on this unit cost information, annual water bills were computed for monthly water consumption of 12,000 gallons per month (45,424.91 liters) via the following formula:

Annual water bill=Per gallon (liter) cost water × 12,000 gallons (45,424.91 liters) × 12 (3)

Table 2 contains the annual water cost for 12,000 gallons (45,424.91 liters) of water consumption per household. Computed annual water bills were verified against existing price information from Circle of Blue, which reports monthly water bills for a sample of U.S. cities for families of four consuming 100 gallons per person per day (378.54 liters), which is comparable to consumption of 12,000 gallons per month (45,424.91 liters). Based on this information, the average annual water bill is approximately $1,686 and the median is $1,620. Thus, the annual water rates used in this paper are somewhat more conservative, but in line, with those outlined by Circle of Blue.

**Table 2:** Affordability Assessment (in 2014 dollars).

	2014 Water Rates	6% Rate Increase	41% Rate Increase
**Water consumption (gallons/month)**	12,000	12,000	12,000
**Monthly Water Bill**	$120	$127.20	$169.20
**Annual Water Bill**	$1,440	$1,526.40	$2,030.40
**Minimum Annual Income to Afford Water Bill**	$32,000	$33,920	$45,120
**Number of Households Below Affordability Benchmark**	13,800,000	17,000,000	40,900,000
**Percent of Households Below Affordability Benchmark**	11.9%	14.7%	35.6%

Affordability is assessed according to the EPA’s affordability benchmarks. Per these criteria, water bills that constitute more than 2% of median household income, and combined water and wastewater bills that constitute more than 4.5% of median household income are considered unaffordable [30]. The emphasis in this study will focus on the combined criteria of 4.5% of income given the rising costs of wastewater treatment, which now comprise a growing portion of water bills [6]. This approach is similar to a recent analysis of water affordability for the state of California [18]. Per the information contained in Table 2 for example, a household would need to make at least $32,000 per year in order to meet EPA’s affordability criteria. To compute the number of households affected, it is possible to find the number of households with median incomes that fall below this affordability threshold.

To understand the distribution of household incomes, and compare this against the benchmark figures provided in Table 2, median income data were obtained by Census Tract from the American Community Survey (ACS) 2010–2014 5 year estimates archived by the National Historic Geographic Information System (NHGIS) [41]. In addition to income data, contextual demographic and socio-economic data which obtained from the ACS via the NHGIS data portal and are defined in Table 3. These data were obtained per the suggestion of prior studies [34], which recommended additional indicators of economic stress be considered to assess the severity of affordability issues, in addition to income based indicators alone. Census tracts are used as the unit of analysis because they approximate neighborhoods within metropolitan areas [42,43].

**Table 3:** Definition of Demographic and Socio-Economic Variables.

Variable	Definition
Percent Disabled	Percentage of the civilian non-institutionalized population with a disability
Median Income White	Median income of the White population in a Census tract.
Median Income Black	Median income of the Black population in a Census tract.
Median Income Hispanic	Median income of the Hispanic population in a Census tract.
Percent Black	Percentage of the tract population that is Black.
Percent Hispanic	Percentage of the tract population that is Hispanic.
Percent Gross Rent	Median gross rent as a percentage of household income
Percent Unemployed	Percent of the civilian population 16 years and older that is unemployed.
Percent No Health Insurance	Percentage of the civilian non-institutionalized population without health insurance
Aggregate Public Assistance Income	Amount of public assistance income received in the last 12 months
Percent Less Bachelor's Degree	Percent of the population 25 years and over without a bachelor's degree or higher
Percent Female Headed Households	Percent of female headed households: no partner present
Percent Food Stamps/SNAP	Percent of households that received food stamps/SNAP in the last 12 months

## Results

Table 2 contains estimates of average annual water rates based on 12,000 gallons (45,424.91 liters) of consumption per month in 2014 dollars. To understand the number of households affected by rising water rates, the median household income (MHI) needed to afford the billing rates in Table 2 was computed. As mentioned previously, this is based on the 4.5% of MHI affordability criterion from the EPA. Households for whom water bills comprise 4.5% or more of MHI face potential affordability issues. To determine the number of households for whom water is unaffordable, a count of the number of households with median incomes below the threshold income provided in Table 2 was tabulated. For example, based on 2014 water rates, 13,756,605 households or 11.9% of all households in the continental United States have incomes below the threshold of $32,000. Households with incomes below this threshold allocate more than 4.5% of their income to pay for water services. This means a household making $25,000 annually, for example, would allocate 6% of their income to pay for water. While this may not seem problematic, this means that these households will have to allocate monies from other expenses to pay for water. Although this may not be a problem for higher income households, this is an issue for low-income and households in poverty who barely make enough money to pay for basic living expenses. Table 2 is valuable because it provides a quick reference, based on current water bill levels, for understanding the potential affordability of water for households of varying income levels.

As described previously, the number of households facing potential affordability challenges was determined by tabulating the number of households below the income threshold necessary for water to be affordable (which is 4.5% or less of MHI). To understand how rising water rates may impact the affordability of water for households in the future, Table 2 also contains projections of water rates based on price increases of 6% and 41% [7]. These projections are based on a 6% rise in water costs between 2014 and 2015, and a rise in water costs of 41% since 2010 [7]. Here, it is important to note that income figures from 2014 are used and not projected to increase over the next five years. This is based on no change or flat trends in household incomes over the last twenty years [44].

Table 2 highlights that if water prices increase more than 6%, which many studies project is the case [8,9], and, which has already happened in some cities [45,46], 17,006,525 households (14.7%) will face affordability challenges. If water prices rise to 41% of 2014 levels, an estimated 35.6% of households will confront affordability issues. This is concerning given the conservative nature of these projections. In comparison, some studies have forecasted that water prices could quadruple in the next 20 years [8]; cities such as Austin, Texas; Charlotte, North Carolina; Chicago, Illinois; San Francisco, California and Tucson, Arizona have all experienced water rate hikes of over 50% in the past five years [6].

These projections are also alarming given the types of people that are likely to be affected by water affordability challenges. To understand the demographic and socio-economic profiles of households facing potential affordability challenges, Census tracts were divided into those with median incomes less than $32,000 and those with median incomes greater than or equal to $32,000. This income threshold is used because it is the household income needed to afford the average water bill for a household of four consuming 12,000 gallons (45,424.91 liters) per month. Next, analysis of variance (ANOVA) was used to determine statistical differences between these two groups of census tracts. To conduct this analysis, separate ANOVA tests were computed for each of the variables listed in Table 3 to determine if the mean value of the variables in tracts with incomes less than $32,000 was statistically different from the mean of tracts whose incomes were greater than or equal to $32,000. Table 4 contains the results and additional statistical information about this analysis including the number of observations, sum of squares between groups and within groups, and the F-statistic. Across all of these demographic and socio-economic characteristics, the two sets of Census tracts are statistically different at the 1% level.

**Table 4:** ANOVA Analysis.

	Mean (Tracts Under $32,000)	Mean (Tracts Income Greater than / equal to $32,000)	Frequency Under $32,000	Frequency Above $32,000	Sum of Squares Between Groups	Sum of Squares Within Groups	Mean Square Between Groups	Mean Square Within Groups	F-Statistic
Percent Disabled	17.3%	12.1%	11,318	60,932	25.83	246.40	25.83	0.003	7,573.75
Median Income White (2014 $)	$25,919	$64,061	11,318	60,932	1.39E+13	4.99E+13	1.39E+13	691339270	20,086.67
Median Income Black (2014 $)	$17,084	$28,801	11,318	60,932	1.31E+12	8.69E+13	1.31E+12	1.20E+09	1,089.43
Median Income Hispanic (2014 $)	$16,272	$40, 240	11,318	60,932	5.48E+12	1.03E+14	5.48E+12	1.42E+09	3,849.38
Percent Black	33.4%	9.7%	11,318	60,932	535.55	2,973.78	535.55	0.04	3,011.17
Percent Hispanic	21.5%	14.5%	11,318	60,932	45.62	3,239.61	45.62	0.04	1,017.39
Percent Gross Rent of Income	35.7%	30.3%	11,318	60,932	2.77E+05	5.72E+06	2.77E+05	79.20	3,501.35
Percent Unemployed	16.3%	8.6%	11,318	60,932	56.63	218.55	56.63	0.003	8,720.02
Percent No Health Insurance	21.4%	13.0%	10,892	60,932	64.58	515.02	64.58	0.01	9,005.44
Aggregate Public Assistance Income (2014 $)	$241,713	$152,444	11,318	60,932	7.61E+13	3.80E+15	7.61E+13	5.27E+10	1,444.29
Percent Less Bachelor's Degree	41.7%	37.5%	10,987	60,932	16.10	1,009.78	16.10	0.01	1,146.34
Percent on Food Stamps	33.0%	10.8%	10,805	60,932	452.86	649.3	452.86	0.009	50,031.84
Percent Female Headed Households	15.6%	4.3%	11,318	60,932	121.71	251.74	121.71	0.003	34,930.32

This table highlights distinct demographic and socio-economic differences between census tracts with median incomes under the required $32,000 and census tracts with median income greater than or equal to $32,000. Tracts with median incomes under $32,000 have higher percentages of persons that are disabled, are without health insurance, and have higher rates of unemployment (Table 4). These low-income tracts also have higher levels of public assistance income, more receipts of food stamps/SNAP assistance, and female sole heads of household. Another noticeable aspect of this table is the prevalence of disabled individuals, as well as concentrations of Blacks and Hispanics. The data highlight that water affordability challenges are perhaps particularly stark for these two groups which have median incomes substantially lower than Whites; the median incomes for White households is $25,919 compared to $16,273 for Hispanic households and $17,085 for African-American households.

Given these stark differences in tracts with median incomes above and below the threshold income of $32,000, a sensitivity analysis was conducted with threshold incomes of $33,920 and $45,120. These benchmarks are used because they represent income thresholds needed to afford the average water bill if water rates rise by 6% and 41%, respectively. The results of this sensitivity analysis are provided in Tables 5 and 6. Results in both tables were statistically significant at the 1% level. These tables highlight that while the differences between tracts below and above the critical income thresholds are smaller than the large differences based on the income threshold of $32,000 presented in Table 4, statistical differences persist. For example, census tracts with median incomes under $45,120 have unemployment rates of 13.1% compared to 7.6% for tracts with median incomes over $45,120. These same tracts also have more people on food stamps; 24.2% compared to 7.6%. Combined, these results suggest tracts falling below the affordability thresholds outlined in this study face additional economic pressures, which reduce their ability to adapt to rising water rates.

**Table 5:** ANOVA Sensitivity Analysis.

	Mean (Median Income Under $33,920)	Mean (Tracts Median Income Greater than/ equal to $33,920)	Frequency Under $33,920	Frequency Above $33,920	Sum of Squares Between Groups	Sum of Squares Within Groups	Mean Square Between Groups	Mean Square Within Groups	F-Statistic
Percent Disabled	17.2%	11.9%	13,515	58,735	30.37	241.85	30.37	0.003	9,072.72
Median Income White (2014 $)	$27,455	$65,135	13,515	58,735	1.56E+13	4.82E+13	1.56E+13	667638717	23,364.47
Median Income Black (2014 $)	$17,527	$29,137	13,515	58,735	1.48E+12	8.67E+13	1.48E+12	1.20E+09	1,233.86
Median Income Hispanic (2014 $)	$17,182	$40,927	13,515	58,735	6.20E+12	1.02E+01	6.20E+12	1.41E+09	4,379.54
Percent Black	31.2%	9.4%	13,515	58,735	522.77	2,986.56	522.77	0.04	12,646.30
Percent Hispanic	21.6%	14.3%	13,515	58,735	59.02	3,226.21	59.02	0.04	1,321.72
Percent Gross Rent of Income	35.6%	30.1%	13,515	58,735	321,800.61	5,677,667.17	321,800.61	78.59	4,094.89
Percent Unemployed	15.8%	8.4%	13,515	58,735	59.20	215.98	59.20	0.003	19,802.83
Percent No Health Insurance	21.3%	12.7%	13,089	58,735	77.95	501.65	77.95	0.01	11,159.74
Aggregate Public Assistance Income (2014 $)	$239,344	$149,650	13,515	58,735	8.84E+13	3.79E+15	8.84E+13	5.25E+10	1,683.78
Percent Less Bachelor's Degree	41.9%	37.3%	13,184	58,735	22.61	1,003.27	22.61	0.01	1,620.57
Percent on Food Stamps	31.5%	10.3%	13,002	58,735	475.99	626.16	475.99	0.01	54,531.34
Percent Female Headed Households	14.8%	4.1%	13,515	58,735	124.39	124.39	124.39	0.00	36,082.01

**Table 6:** ANOVA Sensitivity Analysis.

	Mean (Tracts Median Income Under $45,120)	Mean (Tracts Median Income Greater than/equal to $45,120)	Frequency Under $45,120	Frequency Above $45,120	Sum of Squares Between Groups	Sum of Squares Within Groups	Mean Square Between Groups	Mean Square Within Groups	F-Statistic
Percent Disabled	16.2%	10.7%	28,743	43,507	52.0	220.2	52.0	0.003	17,056.99
Median Income White (2014 $)	$34,898	$73,405	28,743	43,507	2.57E+13	3.82E+13	2.57E+13	5.28E+08	48,576.85
Median Income Black (2014 $)	$19,180	$32,108	28,743	43,507	2.89E+12	8.53E+13	2.89E+12	1.18E+09	2,449.98
Median Income Hispanic (2014 $)	$21,428	$46,433	28,743	43,507	1.08E+13	9.76E+13	1.08E+13	1.35E+09	8,013.47
Percent Black	22.2%	7.7%	28,743	43,507	365.2	3,144.13	365.2	0.044	8,391.92
Percent Hispanic	20.0%	12.7%	28,743	43,507	91.5	3193.7	91.5	0.044	2,070.06
Percent Gross Rent of Income	33.9%	29.3%	28,743	43,507	367,089.88	5,632,377.90	367,089.88	78.0	4,708.76
Percent Unemployed	13.1%	7.6%	28,743	43,507	53.8	221.4	53.8	0.003	17,550.76
Percent No Health Insurance	19.5%	10.9%	28,317	43,507	129.3	450.3	129.3	0.006	20,614.74
Aggregate Public Assistance Income (2014 $)	$210,470	$137,332	28,743	43,507	9.26E+13	3.79E+15	3.79E+15	5.24E+10	1,765.64
Percent Less Bachelor's Degree	42.7%	35.2%	28,412	43,507	95.0	930.9	95.0	0.013	7,339.71
Percent on Food Stamps	24.2%	7.6%	28,230	43,507	472.1	630.1	472.1	0.009	53,746.10
Percent Female Headed Households	10.9%	2.9%	28,743	43,507	109.8	263.6	109.8	0.004	30,093.84

Given these analytical results, which highlight both income-based and contextual demographic and socio-economic pressures on households for whom water affordability is an issue (now and in the future), census tracts are divided into two categories: high-risk and at-risk. The high-risk group is defined as people located in census tracts with median incomes below $32,000. These are census tracts with likely concentrations of people who face affordability challenges based on current water rates. The at-risk group is defined as tracts with median incomes between $32,000 and $45,120. These at-risk tracts have concentrations of people with median incomes below the minimum income thresholds needed to afford future increases in water rates. These at-risk households make up an astonishing 23.5% of all American households. That means an additional 27,181,644 households could soon face challenges in affording basic water and sewer services, if water rates rise by projected or greater than projected amounts.

To better understand the geographic locations of high-risk and at-risk groups, the percentage of all tracts in each group was tabulated by state. This was done to standardize the counts so as not to get an overrepresentation of these groups in states with a large number of census tracts. The rankings of states with overrepresentations of these two groups are based on the percentage of tracks deemed high-risk or at-risk. Tables 7 and 8 contain the percent of high-risk and at-risk tracts by state and their associated rankings. The top five states with the highest percentage of tracts in the high-risk category include Mississippi, Louisiana, Alabama, Kentucky, and Arkansas (Table 7). The top five states with the highest percentage of tracts in the at-risk category include West Virginia, Arkansas, Idaho, Montana, and Mississippi (Table 8).

**Table 7:** Distribution of High-Risk Tracts by State.

State	Number of Tracts	Percent of State's Tracts	Rank
Mississippi	241	36.5%	1
Louisiana	333	29.3%	2
Alabama	334	28.3%	3
Kentucky	310	27.8%	4
Arkansas	187	27.3%	5
Tennessee	370	24.7%	6
West Virginia	110	22.7%	7
South Carolina	247	22.5%	8
Ohio	658	22.3%	9
Georgia	432	22.0%	10
New Mexico	105	21.0%	11
Michigan	579	20.9%	12
Indiana	299	19.8%	13
Arizona	299	19.7%	14
Missouri	270	19.4%	15
North Carolina	411	18.8%	16
Florida	784	18.6%	17
Oklahoma	193	18.5%	18
Texas	968	18.4%	19
District of Columbia	29	16.2%	20
Pennsylvania	487	15.1%	21
New York	716	14.6%	22
Nevada	99	14.4%	23
Illinois	436	14.0%	24
Rhode Island	33	13.7%	25
Kansas	103	13.4%	26
Wisconsin	155	11.1%	27
Idaho	33	11.1%	27
California	837	10.4%	28
Connecticut	84	10.1%	29
Massachusetts	147	10.0%	30
Nebraska	53	10.0%	30
Oregon	78	9.4%	31
Virginia	176	9.3%	32
Maine	32	9.1%	33
South Dakota	19	8.6%	34
Montana	23	8.5%	35
Iowa	65	7.9%	36
Colorado	98	7.8%	37
New Jersey	155	7.7%	38
Utah	38	6.5%	39
Maryland	89	6.4%	40
Washington	88	6.1%	41
Delaware	13	6.0%	42
Minnesota	68	5.1%	43
Vermont	9	4.9%	44
North Dakota	10	4.9%	44
Wyoming	5	3.8%	45
New Hampshire	10	3.4%	46

**Table 8:** Distribution of At-Risk Tracts by State.

State	Number of Tracts	Percent of State's Tracts	Rank
West Virginia	227	46.9%	1
Arkansas	290	42.3%	2
Idaho	119	39.9%	3
Montana	102	37.6%	4
Mississippi	245	37.1%	5
Alabama	429	36.4%	6
Oklahoma	379	36.2%	7
South Carolina	396	36.1%	8
Maine	126	35.9%	9
Tennessee	515	34.4%	10
Kentucky	370	33.2%	11
New Mexico	164	32.9%	12
Missouri	453	32.5%	13
North Carolina	704	32.2%	14
South Dakota	69	31.1%	15
Florida	1297	30.8%	16
Georgia	589	30.0%	17
Oregon	244	29.5%	18
Kansas	221	28.7%	19
Louisiana	320	28.1%	20
Indiana	400	26.5%	21
Texas	1381	26.3%	22
Arizona	392	25.8%	23
Michigan	707	25.6%	24
Ohio	744	25.3%	25
Nebraska	132	24.8%	26
Wisconsin	344	24.7%	27
Iowa	197	23.9%	28
Pennsylvania	709	22.0%	29
Colorado	274	21.9%	30
Nevada	142	20.7%	31
Illinois	645	20.7%	31
Vermont	37	20.1%	32
North Dakota	41	20.0%	33
Virginia	366	19.3%	34
California	1500	18.7%	35
Minnesota	241	18.1%	36
Washington	250	17.3%	37
Wyoming	22	16.7%	38
Rhode Island	40	16.6%	39
Utah	97	16.5%	40
New York	774	15.8%	41
District of Columbia	27	15.1%	42
New Jersey	248	12.4%	43
Delaware	25	11.6%	444
Connecticut	96	11.6%	444
New Hampshire	33	11.2%	45
Massachusetts	162	11.0%	46
Maryland	140	10.0%	47

**Fig 1:**
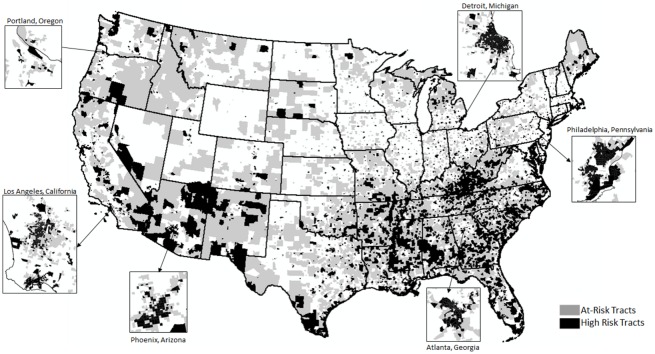
At-Risk and High-Risk Census Tracts.

Fig 1 illustrates the location of these high-risk and at-risk tracts. These tracts are concentrated primarily in urban areas across the country; 81% of high-risk and 63% of at-risk tracts are located in Census-defined urbanized areas. In this figure, clusters of these tracts, or “pockets of water poverty” are highlighted in low-income areas of downtown Detroit, Michigan, downtown Phoenix, Arizona, and downtown Philadelphia, Pennsylvania. Identification of these high-risk areas is particularly critical to understanding and working with utilities in planning for affordability crises, which can occur when a large percentage of consumers are unable to afford water bills. This places dual stresses on these utilities; not only are they facing increasing costs associated with maintaining and upgrading infrastructure, but a shrinking consumer base that is unable to afford these rising costs. If unaffordable water bills from both rising costs and a shrinking population to pay for services cause residents to fall behind on water payments, this can mean the termination of services via water shutoffs. This is not only an economic and public health issue for residents with no service, but an economic issue for utility providers whohave fewer customers over which to spread the large fixed costs of water service. This means affordability issues have cascading impacts for other customers, whose water rates may rise as utilities seek to recover the costs of service by raising rates.

## Discussion

As water rates rise, and household incomes remain stagnant for the foreseeable future, the results of this nationwide assessment highlight a burgeoning affordability crisis for several states, and providers serving low-income households across the nation. The highrisk and at-risk households identified in this study face compounding economic factors that impact their ability to pay for water services. These factors include higher rates of unemployment, lack of health insurance coverage, and a reliance on foods stamps and public assistance. From a geographic perspective, populations most likely to suffer from rising water prices are concentrated in low-income states across the United States. They are also spatially clustered within metropolitan areas across the country, which is likely problematic for utilities who have high numbers of customers in these high-risk and at-risk groups.

Thus, while water rates are currently unaffordable for an estimated 11.9% of households, the conservative estimates of rising rates used in this study highlight that this number could grow to 35.6% in the next five years. More dramatic rate increases could place an even larger segment of the population at-risk. The privatization of water services could also mean much higher water rates for customers. The privatization of water services is one of the factors behind the high water costs in Atlanta, Georgia, which at $325.52 per month has the most expensive water services in the nation [7]. For water to be affordable at these rates, households must make at least $86,805, which is 1.6 times higher than the most recent estimates of U.S. median household income of $53,657 [47].

That said, there are some limitations to this study that are important to note. First, water rates between providers are highly variable within metropolitan areas [22]. Thus, the sample of providers used to compute the average water rate in this study is not necessarily indicative of the water rates that all consumers pay. Second, incomes within census tracts are highly variable which means that the financial ability to pay for water services within metropolitan areas is also highly variable. Third, the study makes use of data from the American Community Survey (ACS), which is subject to a large degree of sampling error. This means that the median income figures used and the number of households that fall above and below this benchmark are variable and could change the percentage of high-risk and at-risk tracts estimated in this study. To determine the sensitivity of analytical results, robustness checks for the state level analysis in Tables 7 and 8 were run with data at higher levels of aggregation (i.e. the county). This analysis revealed that the states with a large number of high-risk and at-risk census tracts were robust to this change in the unit of analysis.

Given the potential impact of rising water rates on a large segment of the population, solutions are needed to resolve this burgeoning affordability issue. Unfortunately, in the United States, assistance programs to help resolve affordability problems for consumers is left to the discretion of individual water providers [10]. A common approach for dealing with delinquent accounts is to shut off water services [5,48–51], and there is little households can do to combat this because there is no federal statute or policy that ensures water access for poor residents [19]. There are also no national standards to protect vulnerable populations (children, the elderly, disabled, and pregnant women) against the termination of water services due to default on payments, nor are there any federal laws or policies governing water affordability [10]. While affordability standards have been adopted by the United Nations and also the U.S EPA, the issue is that none of these standards have any legal framework regarding the enforcement of these standards.

Given this lack of protection for vulnerable populations and other low-income consumers, it has been recommended that federal laws be put into place, similar to those in the United Kingdom, that make it illegal for service providers to disconnect water service due to nonpayment or delinquent payments [10]. In addition to the establishment of laws at the federal and/or state level, mechanisms for reducing the financial burden on households need to be put in place for low-income consumers. While there are a variety of strategies for reducing the financial burden on families, there are four basic types of assistance that could be devised. The first type of assistance would help all water consumers, and that is the financing of infrastructure outlays and improvements by the state and federal government. This approach would reduce the burden on individual providers and reduce the need for increased water rates, because it reduces one of the primary stressors on water rates, rising infrastructure costs. The second type of assistance would involve the subsidization of water services for qualifying low-income households by the local, state, and/or federal government directly. To do this, formal guidelines would need to be developed to determine who qualifies for assistance. Another means of subsidizing low-income households would be the use of community assistance programs to help households pay water bills. In this scenario, non-profit organizations collect and use donations to help households pay their bills [34]. A third type of strategy would involve a restructuring of water rates to reduce the financial burden on low-income consumers. This type of solution, and the best way to structure water rates, has received a lot of attention [17,34,52]. Recent research highlights that rate restructuring is a utility strategy for ensuring cost-recovery of the rising costs of water service [6]. Unfortunately, a rise in the fixed costs or minimum monthly bill for all customers enhances disproportionately the financial burden on low-income households who already face challenges with paying for service [7]. Raising consumer awareness about water use and water costs is a fourth strategy that could be implemented by utilities to help low-income households manage water use and budget for water costs [34]. This type of approach includes a range of options such as consumer counseling, increased frequency of water bills, and the promotion of water conservation strategies to reduce water use [34].

## Conclusion

As a variety of pressures on urban water systems from climate change, suburbanization, shrinking populations in deindustrialized cities, and rising costs of infrastructure grow, a range of actors (governments, utilities, and consumers) will need to work together to solve a growing affordability problem. Water is a fundamental right for all humans [53]. However, a growing number of people globally face daily barriers to accessing clean, affordable water. Thus, it is in the best interest of all people to work to resolve the rising costs of increasingly scarce water resources. This includes utilties who have a vested interest in solving the affordability riddle to mitigate the costs of unaffordable water that include water shut-offs, unpaid accounts, and the time and cost associated with debt collection efforts [8]. The goal of this study was to bring a geographic perspective to this topic in a United States context, which remains a comparatively understudied country in international work on water affordability issues. The hope of this piece is that enhanced awareness of this issue in the developed world will highlight the severity of this issue, which is not isolated to people in the developing world.

## Supporting Information

S1 Table Water Cost Information by Provider. (XLSX)

## References

[1] Banerjee SG, Morella E. Africa’s water and sanitation infrastructure: access, affordability and alternatives [Internet]. Vol. 33, Technical Reports in Hydrology and Water Resources. 2011. 62 p. http://www.un.org/waterforlifedecade/pdf/2006_unwater_coping_with_water_scarcity_eng.pdf\nhttp://www.fhi360.org/NR/rdonlyres/etl7vogszehu5s4stpzb3tyqlpp7rojv4waq37elpbyei3tgmc4ty6dunbccfzxtaj2rvbaubzmz4f/overview1.pdf\nhttp://msf.openrepository.com/msf/ha

[2] Fitch M, Price H. Water poverty in England and Wales. Journal of Poverty and Social Justice. 2002.

[3] Fankhauser S, Tepic S. Can poor consumers pay for energy and water? [Internet]. 2005. Report No.: 92. http://www.ebrd.com/downloads/research/economics/workingpapers/wp0092.pdf

[4] Hunter G. Detroit to start water shut-offs Monday. The Detroit News. 2016.

[5] Spencer G. 7 Years, No Water at Home for Senior [Internet]. 2016. http://www.nbcphiladelphia.com/news/local/7-Years-No-Water-375060031.html

[6] Walton B. Price of Water 2014: Up 6 Percent in 30 Major U.S. Cities; 33 Percent Rise Since 2010 [Internet]. Circle of Blue. 2014. p. 1–12. http://www.circleofblue.org/waternews/2014/world/price-water-2014-6-percent-30-major-u-s-cities-33-percent-rise-since-2010/

[7] Walton B. Price of Water 2015: Up 6 Percent in 30 Major U.S. Cities [Internet]. Circle of Blue. 2015. http://www.circleofblue.org/2015/world/price-of-water-2015-up-6-percent-in-30-major-u-s-cities-41-percent-rise-since-2010/

[8] Baird GM. Water affordability: Who’s going to pick up the check? J / Am Water Work Assoc. 2010;102(12):16–23.

[9] AWWA. Buried No Longer: Confronting America’s Water Infrastructure Challenge. 2012.

[10] Jones P, Moulton A. The Invisible Crisis: Water Unaffordability in the United States [Internet]. 2016. papers2://publication/uuid/99ADF4A4-3045-41CF-A2B2-ACF6167B904E

[11] Hartley D. Urban decline in rust-belt cities [Internet]. Federal Reserve Bank of Cleveland. 2013. http://www.clevelandfed.org/research/commentary/2013/2013-06.cfm

[12] Food & Water Watch. The State of Public Water In the United States [Internet]. 2016. http://www.foodandwaterwatch.org/insight/state-public-water-united-states

[13] Interlandi J. The Race to Buy Up the World’s Water. Newsweek [Internet]. 2010;1–18. http://www.newsweek.com/race-buy-worlds-water-73893

[14] Lappé A. Detroit’s fight for public water is also the nation’s. Al Jazeera English. 2014.

[15] Warner ME. Water privatization does not yield cost savings [Internet]. 2011. http://www.tni.org/tnibook/reclaiming-public-water-2

[16] García-Valiñas M, Martínez-Espiñeira R, González-Gómez F. Affordability of residential water tariffs: Alternative measurement and explanatory factors in southern Spain. J Environ Manage. 2010;91(12):2696–706. doi: 10.1016/j.jenvman.2010.07.029 20709443 10.1016/j.jenvman.2010.07.029

[17] García-Valiñas M, Martínez-Espiñeira R, González-Gómez F. Measuring Water Affordability: A Proposal for Urban Centres in Developed Countries. Int J Water Resour Dev. 2010;26(3):441–58.

[18] U.S. Conference of Mayors. Public Water Cost Per Household : Assessing Financial Impacts of EPA Affordability Criteria in California Cities. 2014.

[19] Carpenter Z. Dry Taps and Lagoons of Sewage: What America’s Water Crisis Looks Like. 2016;1–5.

[20] Reynaud A. Assessing the impact of public regulation and private participation on water affordability for poor households : An empirical investigation of the French case. 2006.

[21] Sebri M. Water affordability and social equity in Tunisian governorates: a distributive approach. Water Policy [Internet]. 2015;17(1):26–45. http://wp.iwaponline.com/content/17/1/26.full

[22] AWWA. 2014 Water and Wastewater Rate Survey. 2015.

[23] Gleick PH. Basic Water Requirements for Human Activities: Meeting Basic Needs. Water Int. 1996;21:83–92.

[24] Chenoweth J. Minimum water requirement for social and economic development. Desalination. 2008;229(1–3):245–56.

[25] White G. Bradley D., and White A., *Drawers of water*: *domestic water use in East Africa*. 1972 The University of Chicago Press. PMC2567632 11884976

[26] Hutton G. Monitoring “Affordability” of water and sanitation services after 2015: Review of global indicator options. 2012;(March 2012):95.

[27] OECD. Social Issues in the Provision and Pricing of Water Services [Internet]. 2003. http://www.oecd-ilibrary.org/environment/social-issues-in-the-provision-and-pricing-of-water-services_9789264099890-en

[28] OECD. Managing Water for All: An OECD Perspective of Pricing and Financing [Internet]. 2009. http://www.partnershipsforwater.net/tc/TC_Tools/105720_OECDManagingWaterforAllAnOECDPerspective.pdf

[29] Sawkins JW, Dickie VA. Affordability of Household Water Services in Great Britain. 2005;26(March):207–13.

[30] U.S. Environmental Protection Agency. Affordability Criteria for Small Drinking Water Systems : An Epa Science Advisory Board Report a Report By the Environmental Economics Advisory Committee of the Epa Science Advisory. 2002;

[31] Detroit Water and Sewerage Deptartment. DWSD Rates Understanding DWSD Water Rates. Detroit; 2006.

[32] United States Government Accountabilty Office. EPA and USDA Are Helping Small Water Utilities with Asset Management ; Opportunities Exist to Better Track Results. 2016.

[33] Duffy M. Challenges In The Water Industry: The Rate Approval Process. 2009.

[34] Beecher JA. Water affordability and alternatives to service disconnection. J / Am Water Work Assoc. 1994;86(10):61–72.

[35] Beecher JA, Shanaghan PE. Water affordability and the DWSRF. J / Am Water Work Assoc. 1998;90(5):68–75.

[36] U.S. Conference of Mayors, American Water Works Association, Water Environment Federation. Affordability Assessment Tool for Federal Water Mandates [Internet]. 2013. http://www.awwa.org/Portals/0/files/resources/waterutilitymanagement/affordability/AffordabilityAssessmentTool.pdf

[37] Beecher JA, Kalmbach JA. 2010 Great Lakes Water Survey. 2011.

[38] Rahill-Marier B, Lall U. America’s Water: An exploratory analysis of Municipal Water Survey Data [Internet]. 2013. http://water.columbia.edu/aquanauts/internships-and-research/americas-water-an-exploratory-analysis-of-municipal-water-survey-data/

[39] AWWA. American Water Works Association: About Us [Internet]. 2016. http://www.awwa.org/about-us.aspx

[40] U.S. Environmental Protection Agency. Indoor Water Use in the United States [Internet]. 2016. https://www3.epa.gov/watersense/pubs/indoor.html

[41] Minnesota Population Center. National Historical Geographic Information System: Version 2.0. Minneapolis, MN: University of Minnesota; 2011.

[42] Darden J, Rahbar M, Jezierski L, Li M, Velie E. The Measurement of Neighborhood Socioeconomic Characteristics and Black and White Residential Segregation in Metropolitan Detroit: Implications for the Study of Social Disparities in Health. Ann Assoc Am Geogr. 2010;100(1):137–58.

[43] Iceland J, Steinmetz E. The Effects of Using Census Block Groups Instead of Census Tracts When Examining Residential Housing Patterns. Soc Forces [Internet]. 2003;(July):1–8. www.census.gov/hhes/www/housing/resseg/pdf/unit_of_analysis.pdf

[44] U.S Bureau of the Census. Real Median Household Income in the United States [Internet]. 2016. https://fred.stlouisfed.org/series/MEHOINUSA672N

[45] San Antonio Water System. 2016 & 2017 Approved Rates Target Long-term Needs [Internet]. 2016. http://www.saws.org/rates/

[46] San Diego City Council. Notice of Public Hearing for Proposed Water Rate Increases [Internet]. 2015. https://www.sandiego.gov/sites/default/files/legacy/water/pdf/rates/151117hearing.pdf

[47] DeNavas-Walt C and, Proctor BD. Income and Poverty in the United States: 2013 [Internet]. Washington D.C.; 2015. https://www.census.gov/content/dam/Census/library/publications/2014/demo/p60-249.pdf

[48] Hager J. Baltimore now shutting off water customers with overdue bills [Internet]. 2015. http://www.abc2news.com/news/region/baltimore-city/baltimore-now-shutting-off-water-to-overdue-businesses

[49] Naylor Gesick J. Idlewild Inn apartments face water shut-off again [Internet]. Rapid City Journal. 2016 http://rapidcityjournal.com/news/local/idlewild-inn-apartments-face-water-shut-off-again/article_a7573dac-5808-5c0e-bc6f-5f7b16cedfe1.html

[50] Wells K. 1,800 homes had water turned off after shutoffs resume in Detroit [Internet]. 2016. http://michiganradio.org/post/1800-homes-had-water-turned-after-shutoffs-resume-detroit

[51] Walton B. Water Systems Need Investment and Affordability [Internet]. Circle of Blue. 2016. http://www.circleofblue.org/2016/water-management/infrastructure/water-systems-need-investment-affordability/

[52] Barberán R, Arbués F. Equity in domestic water rates design. Water Resour Manag. 2009;23(10):2101–18.

[53] United Nations General Assembly. Resolution. A/RES/64/292 2010.

