# Correction: A Burgeoning Crisis? A Nationwide Assessment of the Geography of Water Affordability in the United States

**DOI:** 10.1371/journal.pone.0176645

**Published:** 2017-04-21

**Authors:** Elizabeth A. Mack, Sarah Wrase

In Fig 1, the vertical orientation of the inset for Detroit, Michigan is incorrectly reversed. Please see the correct Fig 1 here.

**Fig 1:**
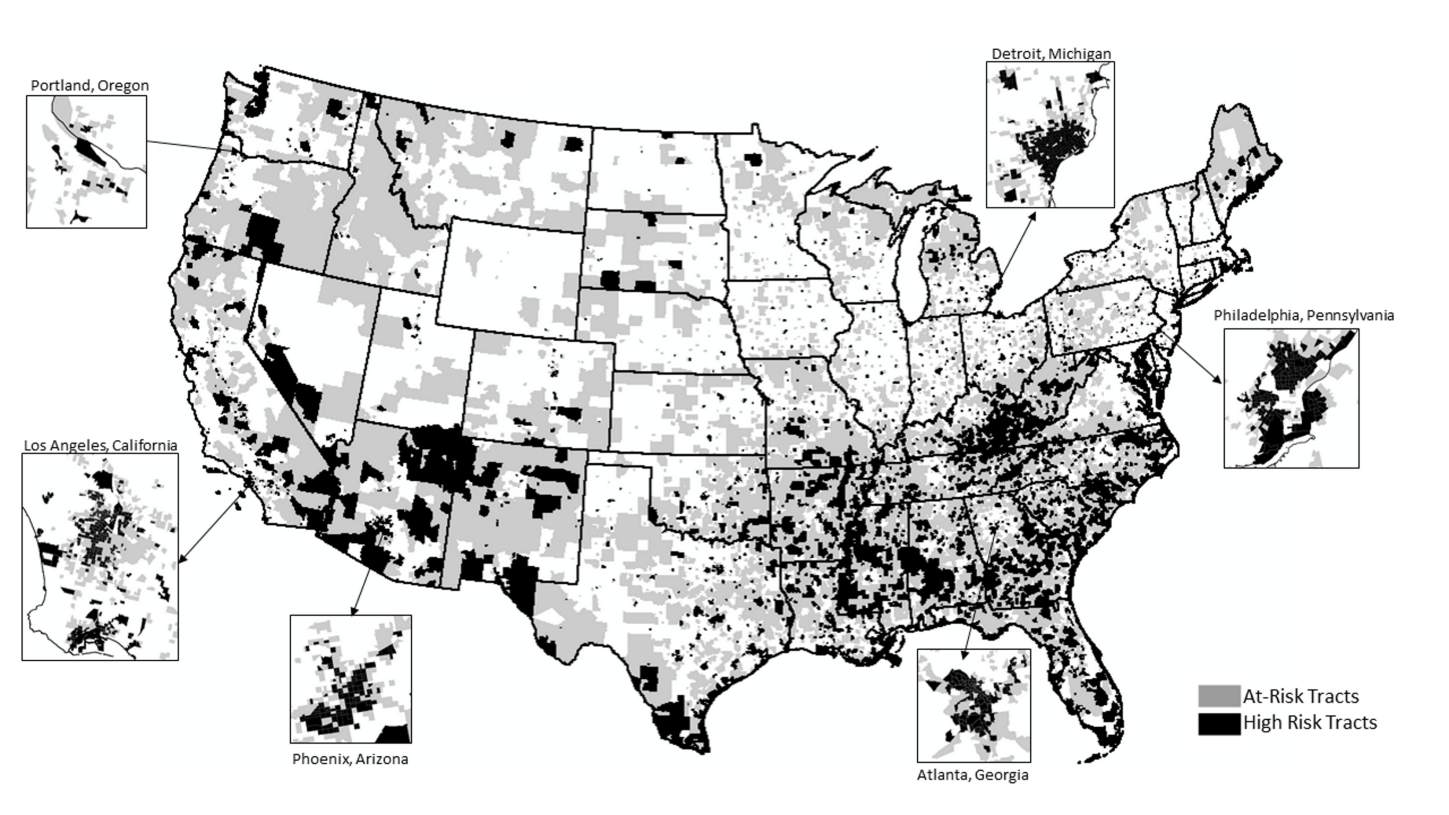
At-Risk and High-Risk Census Tracts.
